# Intermittent fasting on health, aging and disease: what about sleep?

**DOI:** 10.5935/1984-0063.20190152

**Published:** 2020

**Authors:** Miguel Meira e Cruz

**Affiliations:** 1 Cardiovascular Center of University of Lisbon, Lisbon School of Medicine, Sleep Unit -Lisbon - Portugal.; 2 Faculdade São Leopoldo Mandic, Laboratory of Neuroimmune Interface of Pain Research - Campinas - São Paulo - Brazil.

Impact of intermittent fasting (IF) on health, aging and disease was recently revisited by de Cabo et al.^1^ focusing on the evidence from preclinical and clinical trials showing a broad-spectrum benefit on several health domains. Although a highly relevant and interesting critical discussion was conducted, sleep as a major factor interfering on several cardiovascular and metabolic pathways^2^ directly and indirectly related with those profits seems to have been forgotten.

It is worth of note that IF regimens are circadian based physiological challenges, which therefore interfere with sleep-wake cycle^3^, but sleep was not previously explored in the context of IF. Yet, sleep is crucial for metabolic regulation either on health or disease and for example, Obstructive Sleep Apnea as a main representative of the most prevalent sleep disturbances, have been related to impaired glucose and lipid metabolism opening plausibility on its interference on fasting related dietary regimens^4^.

For most the supporters of IF regimens, the fasting period occurs during sleep, meaning that feeding is restricted to daytime. Ramadan, an old Muslim ritual occurring in a different season every 9 years, include a 1-month diurnal IF, with a mealtime confined to the period from dawn to sunset and was taken as a model for studying the effects of IF. Interestingly, current evidence shows that circadian changes (shift delay) associated to time-restricted feeding during Ramadan are not only related to the shift in the mealtime but also to the changes in the sleep patterns usually observed in that season^5^. Meanwhile, not only meal timing is a key-factor in the regulation of circadian timing system and sleep^6^, but also sleep impairment could influence metabolic related mechanisms^7^ which are mainly related to such benefits observed in IF schemes of dietary intake. This should be taken into consideration and sleep-IF interaction should be further explored in future studies, since either IF and sleep related mechanisms could both independently and synergistically contribute to such advantages.
